# Machine Learning Prediction of Pharmacogenetic Testing Uptake Among Opioid-Prescribed Patients Using Electronic Health Records: Retrospective Cohort Study

**DOI:** 10.2196/81048

**Published:** 2026-01-21

**Authors:** Mohammad Yaseliani, Je-Won Hong, Jiang Bian, Larisa Cavallari, Julio D Duarte, Danielle Nelson, Wei-Hsuan Lo-Ciganic, Khoa Anh Nguyen, Md Mahmudul Hasan

**Affiliations:** 1 Department of Pharmaceutical Outcomes and Policy, College of Pharmacy, University of Florida, 1889 Museum Road, Malachowsky Hall, Suite 6300, Gainesville, FL, 32611, United States, 1 352-273-6276; 2 Department of Pharmacotherapy and Translational Research, College of Pharmacy, University of Florida, Gainesville, FL, United States; 3 Department of Biostatistics and Health Data Science, School of Medicine, Indiana University, Indianapolis, IN, United States; 4 Center for Biomedical Informatics, Regenstrief Institute, Indianapolis, IN, United States; 5 Center for Pharmacogenomics and Precision Medicine, University of Florida, Gainesville, FL, United States; 6 College of Medicine, University of Florida, Gainesville, FL, United States; 7 Division of General Internal Medicine, School of Medicine, University of Pittsburgh, Pittsburgh, PA, United States; 8 Center for Pharmaceutical Policy and Prescribing, University of Pittsburgh, Pittsburgh, PA, United States; 9 Geriatric Research Education and Clinical Center, North Florida/South Georgia Veterans Health System, Gainesville, FL, United States; 10 Department of Information Systems and Operations Management, Warrington College of Business, University of Florida, Gainesville, FL, United States

**Keywords:** ensemble model, ML, machine learning, opioids, opioid prescription, pain management, pharmacogenetic testing

## Abstract

**Background:**

Opioids are a widely prescribed class of medication for pain management. However, they have variable efficacy and adverse effects among patients, due to the complex interplay between biological and clinical factors. Pharmacogenetic testing can be used to match patients’ genetic profiles to individualize opioid therapy, improving pain relief and reducing the risk of adverse effects. Despite its potential, the pharmacogenetic testing uptake (use of pharmacogenetic testing) remains low due to a range of barriers at the patient, health care provider, infrastructure, and financial levels. Since testing typically involves a shared decision between the provider and patient, predicting the likelihood of a patient undergoing pharmacogenetic testing and understanding the factors influencing that decision can help optimize resource use and improve outcomes in pain management.

**Objective:**

This study aimed to develop machine learning (ML) models, identifying patients’ likelihood of pharmacogenetic uptake based on their demographics, clinical variables, medication use, and social determinants of health.

**Methods:**

We used electronic health record data from a single center health care system to identify patients prescribed opioids. We extracted patients’ demographics, clinical variables, medication use, and social determinants of health, and developed and validated ML models, including a neural network, logistic regression, random forest, extreme gradient boosting (XGB), naïve Bayes, and support vector machines for pharmacogenetic testing uptake prediction based on procedure codes. We performed 5-fold cross-validation and created an ensemble probability-based classifier using the best-performing ML models for pharmacogenetic testing uptake prediction. Various performance metrics, uptake stratification analysis, and feature importance analysis were used to evaluate the performance of the models.

**Results:**

The ensemble model using XGB and support vector machine–radial basis function classifiers had the highest *C*-statistics at 79.61%, followed by XGB (78.94%), and neural network (78.05%). While XGB was the best-performing model, the ensemble model achieved a high accuracy (32,699/48,528, 67.38%), recall (537/702, 76.50%), specificity (32,162/47,826, 67.25%), and negative predictive value (32,162/32,327, 99.49%). The uptake stratification analysis using the ensemble model indicated that it can effectively distinguish across uptake probability deciles, where those in the higher strata are more likely to undergo pharmacogenetic testing in the real world (320/4853, 6.59% in the highest decile compared to 6/4853, 0.12% in the lowest). Furthermore, Shapley Additive Explanations value analysis using the XGB model indicated age, hypertension, and household income as the most influential factors for pharmacogenetic testing uptake prediction.

**Conclusions:**

The proposed ensemble model demonstrated a high performance in pharmacogenetic testing uptake prediction among patients using opioids for pain. This model can be used as a decision support tool, assisting clinicians in identifying patients’ likelihood of pharmacogenetic testing uptake and guiding appropriate decision-making.

## Introduction

Opioid prescribing presents a complex therapeutic challenge due to variable efficacy and the risk of adverse effects, often resulting in a trial and error approach for prescription. These variations in opioid response often arise from interindividual differences in pharmacokinetics and pharmacodynamics, influenced in part by genetic variations [1]. Over recent decades, pharmacogenetic testing has emerged as a promising strategy to tailor opioid therapy to an individual genetic profile, with the goal of enhancing pain relief, minimizing adverse effects, and improving overall patient outcomes. Several opioids, including codeine, tramadol, hydrocodone, and oxycodone, are metabolized to varying extents by cytochrome P450 family 2 subfamily D member 6 (CYP2D6), which is an enzyme involved in the metabolism of many drugs [2]. A pragmatic trial demonstrated improved composite pain intensity outcomes with CYP2D6-guided pain management, highlighting the potential benefits of personalized therapy based on pharmacogenetic testing [2]. Based on the current evidence, the Clinical Pharmacogenetics Implementation Consortium (CPIC) provides guidelines on using CYP2D6 genotype results for prescribing tramadol, hydrocodone, and codeine [2]^2^ yet their real-world impact depends on whether patients offered pharmacogenetic testing actually complete it. In opioid prescribing contexts, uptake hinges on perceived use and timing, out-of-pocket costs, health literacy, trust, social context, and clinician offering practices, as well as workflow constraints that differ across acute, perioperative, and chronic pain settings.

Electronic health records (EHRs) contain rich information relevant to testing acceptance: prior and current opioid exposures, pain diagnoses, comorbidity burden, care setting, prescriber specialty, monitoring and follow-up patterns, previous laboratory or genetic testing, and engagement with the health system. Machine learning (ML) such as neural network (NN), random forest (RF), and logistic regression (LR) can synthesize these heterogeneous and high-dimensional features [3] to produce calibrated, patient-level probability estimates of pharmacogenetic testing uptake at the moment testing is offered. Such predictions could help health systems, including patient care teams and insurers, tailor communication (eg, language, framing, and channel), select appropriate clinical checkpoints (such as before initiating certain opioids or at dose escalation), and allocate resources where they are most likely to improve test uptake without adding unnecessary burden. These predictions may be especially valuable for patients who are expected to benefit most from testing (though this was not the focus of this study), enabling patient care teams to dedicate additional resources to support uptake in this population.

Prior work has developed ML predictive models to support uptake for vaccines [4], cancer screening [5], and genetic counseling [6]; however, pharmacogenetics-specific models for opioid-related testing are scarce, and few studies evaluate calibration, clinical use, fairness, and workflow integration together. In this study, we aimed to predict the likelihood that patients receiving opioid prescriptions will undergo pharmacogenetic testing after an offer in routine care. We hypothesized that ML models could accurately predict the likelihood of pharmacogenetic testing uptake among opioid-prescribed patients by identifying patterns in demographic, clinical, and health care use factors associated with testing behavior under current practice conditions. Our objectives were to achieve strong discrimination and predictive performance of the ML models, and reliable model calibration through patients’ stratification in subgroups based on the predicted probability of test uptake. We emphasized model explainability to support clinician understanding and sought to identify factors influencing the likelihood of pharmacogenetic testing uptake in opioid therapy. Ultimately, we aimed to complement, rather than replace, clinical judgment, potentially facilitating better use of pharmacogenetic testing for precision pain management. By focusing on actionable predictions at the point of offer, this paper may help programs deliver the right message through the right channel at the right time: improving test uptake, informing safer opioid prescribing, and advancing equitable delivery of pharmacogenetic testing.

## Methods

### Overview

**Figure 1.**
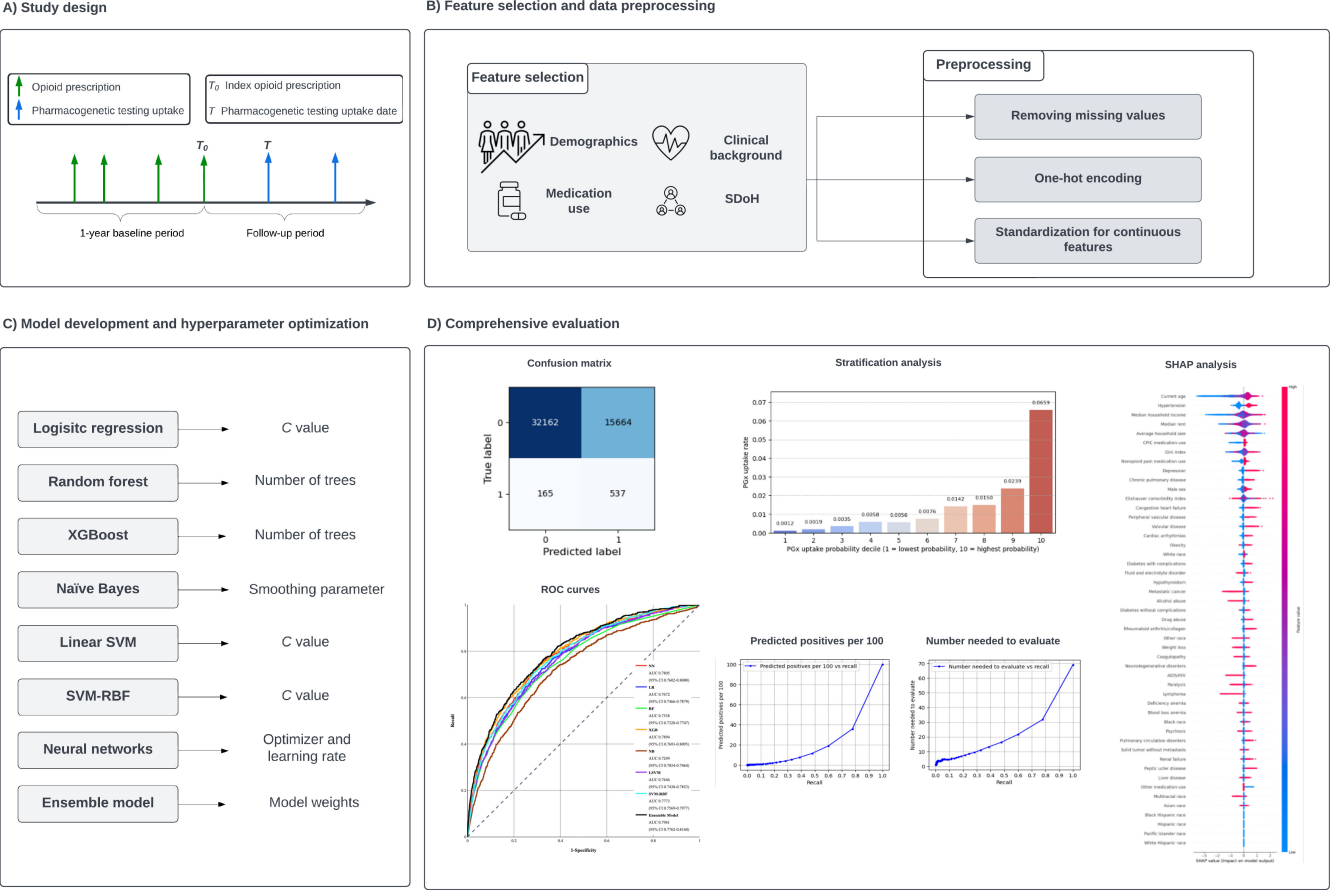
Overall machine learning pipeline for pharmacogenetic testing uptake prediction. CPIC: Clinical Pharmacogenetics Implementation Consortium; ROC: receiver operating characteristic; SDoH: social determinants of health; SHAP: Shapley Additive Explanations; SVM: support vector machine; SVM-RBF: support vector machine–radial basis function; XGBoost: extreme gradient boosting.

The overall ML pipeline for pharmacogenetic testing uptake prediction is shown in Figure 1. The pipeline involves 4 steps, including study design, data preprocessing and feature selection, model development, and comprehensive evaluation. Each step involves several tasks that are critical to building and evaluating the final predictive model. The details of each step are described in this section.

### Ethical Considerations

We used real-world deidentified EHR data available from the University of Florida Health Integrated Data Repository (IDR) to develop the models. These data included information on patients’ demographics, clinical variables, and medication use from 2011 to 2023. The study was conducted in compliance with ethical standards and was approved by the University of Florida Institutional Review Board under the reference number IRB202301927. This study is a secondary analysis of existing deidentified EHR data, and the approved IRB covers secondary analyses without additional consent. Therefore, no informed consent was obtained.

### Study Design

Patients’ age range was 1 to 89 years, and we did not exclude any patients based on age. Patients were included if they were aged between 18 and 89 years and had an opioid prescription order for noncancer treatment from 2011 to 2023. Patients were stratified into an intervention group (patients with a minimum of one pharmacogenetic testing order) and a control group (those with no history of pharmacogenetic testing order). We used current procedural terminology codes to determine patients’ pharmacogenetic testing. To create the cohort for ML model development, we defined the index dates separately for intervention and control groups. The index date for the intervention group was defined as the most recent date of an opioid prescription prior to pharmacogenetic testing. The index date for the control group was defined as the earliest date of opioid prescription. All baseline covariates were collected in the 1-year period prior to the index date. The outcome was a binary target variable, indicating whether the patient was in the intervention or control group based on their pharmacogenetic testing record. If a patient was in the intervention group, their outcome value was encoded as 1 and otherwise 0.

### Feature Selection and Data Preprocessing

We selected four different categories of features for our study sample: (1) demographics, (2) clinical history, (3) medication use, and (4) social determinants of health (SDoH). Demographics included age, sex, and race, where age was continuous, sex was binary, and race was multicategory which was one-hot-encoded to get separate binary inputs for each racial group and prevent the ordinality assumption by the model. We used the *International Classification of Diseases, Ninth Revision* (*ICD-9*) and *International Classification of Diseases, Tenth Revision* (*ICD-10*) codes to determine the clinical history variables, including a diagnosis of AIDS/HIV, alcohol abuse, blood loss anemia, cardiac arrhythmias, chronic pulmonary disease, coagulopathy, systolic (congestive) heart failure, deficiency anemia, depression, diabetes with complications, diabetes without complications, drug abuse, fluid and electrolyte disorder, hypertension, hypothyroidism, liver disease, lymphoma, metastatic cancer, neurodegenerative disorders, obesity, paralysis, peptic ulcer disease, peripheral vascular disease, psychosis, pulmonary circulation disorders, renal failure, rheumatoid arthritis or collagen, solid tumor without metastasis, valvular disease, and weight loss. Furthermore, we included the Elixhauser comorbidity index [7] to calculate the overall health care burden for each patient. In addition, the SDoH information included the average household size, Gini index as a measure of income inequality, median household income, and median gross rent, all linked to the University of Florida Health EHR based on year and ZIP code using the Agency for Healthcare Research and Quality SDoH database [8]. We removed all the missing data, so all patients had complete information for model development.

### Model Development and Hyperparameter Optimization

We adopted a comprehensive strategy for model development using LR, extreme gradient boosting (XGB) [9], RF [10], support vector machine (SVM) [11] with linear and radial basis function (RBF) kernels (ie, linear support vector machine [LSVM] and SVM-RBF), and NN to capture predictive performance across a wide range of models for pharmacogenetic testing uptake prediction. LR was first developed due to its ability to capture linear relationships in the data and providing baseline performance. Similarly, the naïve Bayes (NB) model was developed due to its simplicity and effectiveness in high-dimensional spaces. However, considering LR’s and NB’s limitations in capturing complex nonlinear relationships, we trained tree-based models, including XGB and RF, given their ability to capture nonlinear interactions that may be critical to accurately predicting the pharmacogenetic testing uptake. LSVM and SVM-RBF were developed to capture high-dimensional complex interactions among input features. Finally, the NN was trained to explore more complex interactions among features that may be missed by traditional models.

To train the models, we randomly partitioned the data with a stratified 80/20 train/test split. To mitigate the risk of overfitting and get the best performance out of ML models, we conducted 5-fold cross-validation. For LR, we tuned the hyperparameter *C* with values of 0.01, 0.1, 1, and 10. To tune NB, we used smoothing parameters ranging from 10^−9^ to 10^−5^ with 10x increments. Both XGB and RF models were tuned with 100, 200, 300, 400, and 500 trees to explore the effect of different numbers of trees on performance. LSVM and SVM-RBF were optimized with *C* values equal to 0.01, 0.1, 1, and 10. Finally, NNs were trained with all possible combinations of various optimizers and learning rates. The optimizers included stochastic gradient descent [12], Adam [13], and Nadam [14], and learning rates ranged from 10^−5^ to 10^−1^ with 10x increases. The best hyperparameters were selected based on the highest area under the receiver operating characteristic curve (AUC) or *C*-statistics achieved on the training set during the cross-validation process.

We trained all the models using the best-performing hyperparameters and evaluated their performance on the test set. Notably, we used a balanced class weighting approach, assigning proportionally higher weights to the minority class, to decrease the risk of overfitting when training each of the models. As an additional analysis, we used the synthetic minority over-sampling technique (SMOTE) [15] with a 1:1 class ratio between minority and majority samples, creating a balanced dataset for model training and evaluation. To improve the performance and enhance generalizability of the models, we created a weighted probability-based ensemble classifier. We used a grid search optimization procedure, searching for the best combination of model weights, conditioned on having a total sum of weights equal to 1. The ensemble model achieving the highest AUC or *C*-statistics was selected as the final best model for pharmacogenetic testing uptake prediction.

### Comprehensive Evaluation

We followed TRIPOD+AI (Transparent Reporting of a Multivariable Prediction Model for Individual Prognosis or Diagnosis + Artificial Intelligence) guidelines [16] to evaluate the performance of ML models. AUC or *C*-statistics demonstrates the discriminative ability of the model, considering the trade-off between sensitivity and specificity at different threshold values. DeLong test [17,18] is used to identify whether there is a statistically significant difference between AUC values. Accuracy provides the overall performance of the model by calculating the proportion of true negative and true positive cases compared to all the samples in the data. However, this measure may be misleading for rare events and not suitable for highly imbalanced datasets. To address this, we calculated specificity, which measures the model’s ability to correctly identify true negative cases and recall or sensitivity, indicating the model’s performance for identifying true positive cases. Since model performance may not be optimal, we used the Youden index [19-21] to obtain the best performing classification threshold for each model, where the highest value of “specificity+sensitivity-1” is achieved. To further evaluate model performance for real-world applications, we calculated the number needed to evaluate and predictive positives per 100 patients, showing how many predictions must be made to identify 1 actual positive case and the number of pharmacogenetic testing uptake predictions per 100 individuals, respectively. Moreover, we did stratification analysis, where pharmacogenetic testing uptake probabilities were categorized into 10 deciles in ascending order and the percentage of actual pharmacogenetic testing uptake in the data was identified in each decile to evaluate the ability of the model in identifying more uptakes in the higher strata. In addition, we used the Shapley Additive Explanations (SHAP) [22] analysis to report feature importance and identify the features that are most influential to pharmacogenetic testing uptake.

## Results

### Patient Characteristics

We used data from the University of Florida Health IDR with patients from a wide range of demographic and clinical backgrounds. The data included a total of 455,773 patients, 7645 (1.68%) with a recorded pharmacogenetic testing and 448,128 (98.32%) without. After removing 160,914 further patients who had no opioid prescription, there were 294,859 patients using opioids in the cohort. We further excluded patients with no race information and SDoH variables (after matching with the Agency for Healthcare Research and Quality database). Overall, the final cohort included 242,640 patients, where 3510 (1.45%) had a recorded pharmacogenetic testing uptake and 239,130 (98.55%) did not. The cohort had an average age of 58 (SD 17.47) years, a gender distribution comprising 41.49% (100,670/242,640) as male participants and 58.51% (141,970/242,640) as female participants, and a racial distribution of 64.94% (157,567/242,640) White individuals, 28.77% (69,810/242,640) Black individuals, and 6.29% (15,263/242,640) belonging to other racial groups (ie, Hispanic, White Hispanic, Black Hispanic, Asian, Pacific Islander, American Indian, Multiracial, and other). In addition, the cohort had an average household size of 2.58 (SD 0.28), a Gini index of 0.45 (SD 0.05), and an average median income of US $46,324. Table 1 presents the key sociodemographic characteristics of patients in the cohort. Moreover, the distribution of patient characteristics in the training and test sets is provided in Table 2.

**Table 1.** Sociodemographic summary of patients.

Characteristics	Pharmacogenetic testing	Nonpharmacogenetic testing	*P* value^a^
Number of individuals, n (%)	3510 (1.45)	239,130 (98.55)	—^b^
Sex, n (%)			<.001
Male	1627 (46.35)	99,043 (41.42)	
Female	1883 (53.65)	140,087 (58.58)	
Race, n (%)			<.001
American Indian	12 (0.34)	346 (0.14)	
Asian	30 (0.85)	2345 (0.98)	
Black	1010 (28.77)	68,800 (28.77)	
Black Hispanic	0 (0.00)	1 (0.00)	
Hispanic	1 (0.03)	168 (0.07)	
Multiracial	13 (0.37)	1165 (0.49)	
Pacific Islander	0 (0.00)	109 (0.05)	
White	2346 (66.84)	155,221 (64.91)	
White Hispanic	0 (0.00)	45 (0.02)	
Other	98 (2.79)	10,930 (4.57)	
Age (y), mean (SD)	66.04 (12.99)	58.24 (17.51)	<.001
Average household size, mean (SD)	2.60 (0.27)	2.58 (0.28)	<.001
Gini index^c^, mean (SD)	0.454 (0.051)	0.451 (0.052)	<.001
Median household income (US $), mean (SD)	46,093.44 (15,015.22)	46,328.29 (15,538)	.374
Median gross rent^d^, mean (SD)	898.12 (213.45)	905.81 (223.60)	.034

a *P* values were calculated using a chi-square test for categorical variables and independent 2-tailed* t* test with equality of variance analysis for continuous variables.

b Not applicable.

c The Gini index ranges from 0 to 1, where 0 represents perfect equality and 1 represents perfect inequality.

d The median gross rent is per month in US $ and is based on rented cash-paid housing units.

**Table 2.** Distribution of patient characteristics in the training and test sets.

Characteristics	Training (n=194,112)	Testing (n=48,528)
Sex, n (%)		
Male	80,519 (41.48)	20,151 (41.52)
Female	113,593 (58.52)	28,377 (58.48)
Race, n (%)		
American Indian	273 (0.14)	85 (0.18)
Asian	1877 (0.97)	498 (1.03)
Black	55,993 (28.85)	13,817 (28.47)
Black Hispanic	1 (0.00)	0 (0.00)
Hispanic	128 (0.07)	41 (0.08)
Multiracial	919 (0.47)	259 (0.53)
Pacific Islander	89 (0.05)	20 (0.04)
White	125,938 (64.88)	31,629 (65.18)
White Hispanic	33 (0.02)	12 (0.02)
Other	8861 (4.56)	2167 (4.47)
Age, mean (SD)	58.36 (17.48)	58.32 (17.45)
Average household size, mean (SD)	2.58 (0.28)	2.58 (0.28)
Gini index, mean (SD)	0.45 (0.05)	0.45 (0.05)
Median household income (US $), mean (SD)	46,327.05 (15,529.07)	46,316.28 (15,536.70)
Median rent (US $), mean (SD)	905.64 (223.38)	905.92 (223.78)

### Model Performance

**Figure 2.**
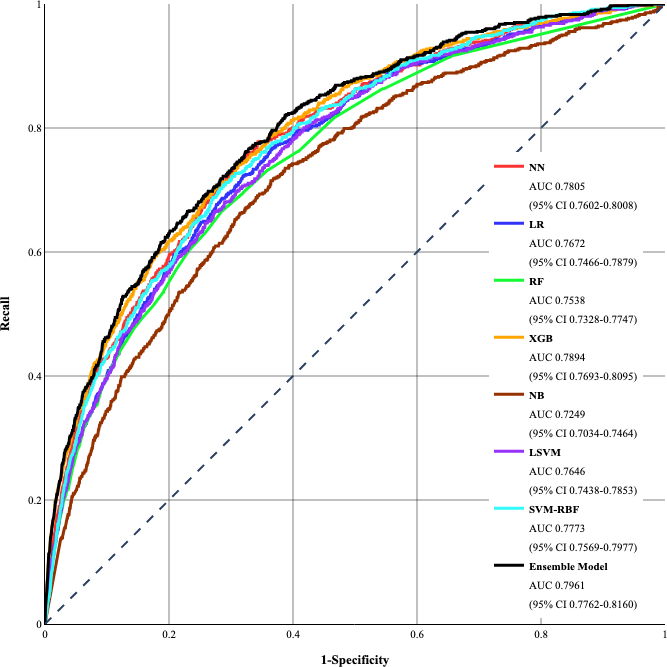
Receiver operating characteristic curves of developed models. AUC: area under the receiver operating characteristic curve, LR: logistic regression, LSVM: linear support vector machine, NB: naïve Bayes, NN: neural network, RF: random forest, SVM-RBF: support vector machine–radial basis function, XGB: extreme gradient boosting.

The ROC curves of all models and their corresponding AUC or *C*-statistics with 95% CIs are provided in Figure 2. The ensemble model achieved the highest *C*-statistics of 79.61% using 0.7 and 0.3 weights for XGB and SVM-RBF, respectively. In contrast, NB achieved the lowest *C*-statistics at 72.49%. Other models had comparable *C*-statistics, equal to 75.38% (RF), 76.72% (LR), 76.46% (LSVM), 77.73% (SVM-RBF), 78.05% (NN), and 78.94% (XGB). Additionally, the DeLong test results indicated that there was a statistically significant difference (*P*<.05) between the *C*-statistics of the ensemble model and all other classifiers, showing its higher performance is less likely to be due to chance.

**Figure 3.**
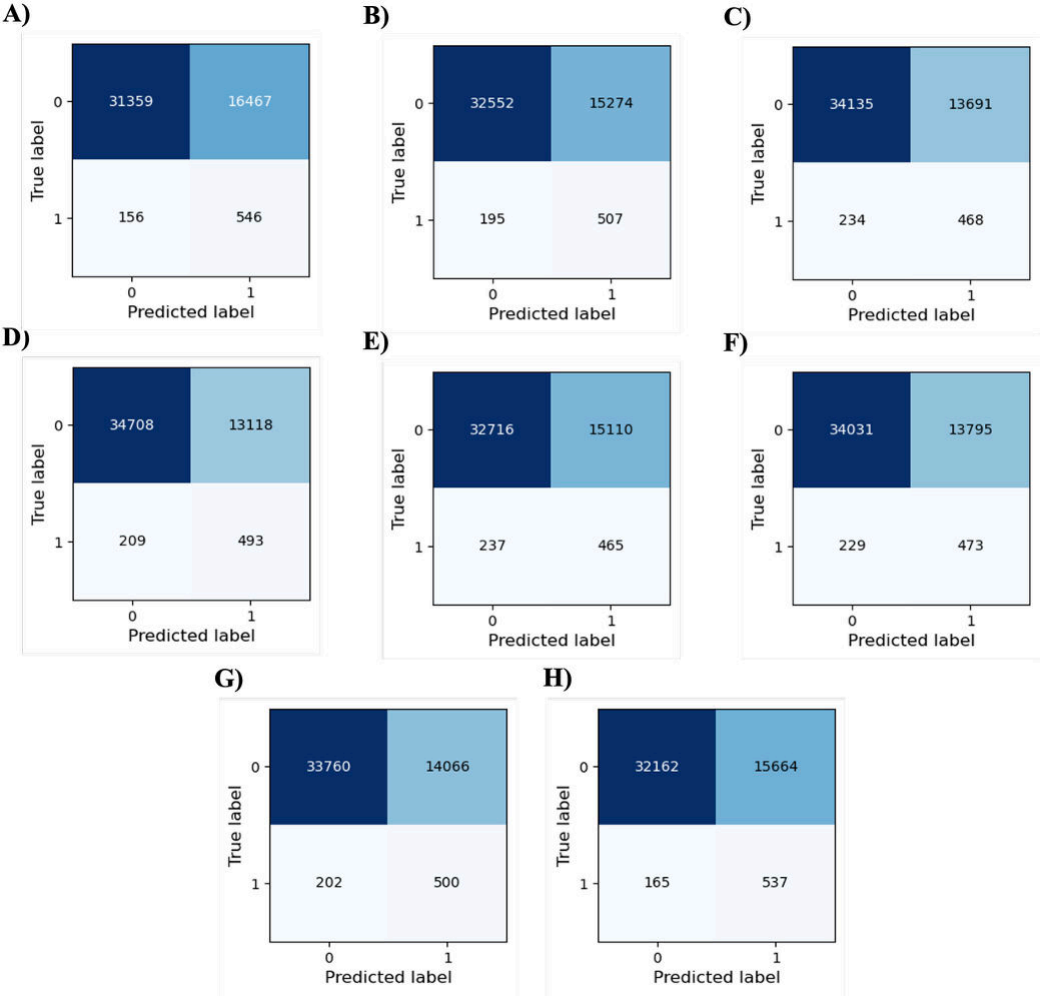
Confusion matrix of models based on Youden index: (A) neural network; (B) logistic regression; (C) random forest; (D) gradient boosting; (E) naïve Bayes; (F) linear support vector machine; (G) support vector machines-radial basis function; and (H) Ensemble model.

Since model performance was not optimal, we used the Youden index [19-21] to obtain the best performing classification threshold for each model. Figure 3 portrays the confusion matrices for all developed models based on their Youden index threshold. Table 3 provides a summary of performance for all developed models. Accuracy representing the proportion of correctly classified cases was highest for XGB (35,201/48,528, 72.54%), followed by RF (34,603/48,528, 71.31%), LSVM (34,504/48,528, 71.10%), and SVM-RBF (34,260/48,528, 70.60%). Other classifiers had 65% to 70% accuracy with the ensemble model having an accuracy of 67.38% (32,699/48,528).

**Table 3.** Performance metrics of developed models based on Youden index.

Model	Accuracy	Recall	Specificity	NPV^a^	PPV^b^	AUC^c^
NN^d^	65.75	77.78	65.57	99.51	3.21	78.05
LR^e^	68.12	72.22	68.06	99.40	3.21	76.72
RF^f^	71.31	66.67	71.37	99.32	3.31	75.38
XGB^g^	72.54	70.23	72.57	99.40	3.62	78.94
NB^h^	68.38	66.24	68.41	99.28	2.99	72.49
LSVM^i^	71.10	67.38	71.16	99.33	3.32	76.46
SVM-RBF^j^	70.60	71.23	70.59	99.41	3.43	77.73
Ensemble	67.38	76.50	67.25	99.49	3.31	79.61

a NPV: negative predictive value.

b PPV: positive predictive value.

c AUC: area under the receiver operating characteristic curve.

d NN: neural network.

e LR: logistic regression.

f RF: random forest.

g XGB: extreme gradient boosting.

h NB: naïve Bayes.

i LSVM: linear support vector machine.

j SVM-RBF: support vector machine–radial basis function.

To assess the model performance in identifying true positive cases (ie, those with a recorded pharmacogenetic testing uptake) and true negative cases (ie, those without a recorded pharmacogenetic testing uptake), recall and specificity were calculated. Recall values ranged from 66.24% (465/702) to 77.78% (546/702) with the NN model achieving the highest recall and the ensemble model achieving 76.50% (537/702). Specificity values ranged from 65.57% (31,359/47,826) to 72.57% (34,708/47,826), with the XGB model achieving the highest specificity. To further assess models’ performance in distinguishing between true positives and true negatives, negative predictive value (NPV) and positive predictive value (PPV) were calculated. NPV values ranged from 99.28% (32,716/32,953) to 99.51% (31,359/31,515) with the NN model having the highest NPV, while PPV values ranged from 2.99% (456/15,575) to 3.62% (493/13,611) with XGB achieving the highest value. To evaluate model fairness, we used the ensemble model to calculate the performance metrics across different racial groups (Table 4). All the metrics, except NPV, exhibited notable variations across different racial groups with AUC ranging from 74.31% to 83.29%, and accuracy from 65.11% (20,592/31,629) to 79.15% (205/259).

**Table 4.** Performance metrics of developed models across racial groups^a^.

Race group	Accuracy	Recall	Specificity	NPV^b^	PPV^c^	AUC^d^
White	65.11	75.27	64.95	99.44	3.11	77.73
Black	70.34	81.59	70.18	99.61	3.88	83.29
Multiracial	79.15	75.00	79.22	99.51	5.36	74.31
Asian	74.90	66.67	75.00	99.46	3.15	82.69
Other	78.27	60.87	78.45	99.47	2.94	82.32

a The performance metrics were reported using the testing data for the racial groups with at least 4 samples in the positive class (ie, pharmacogenetic testing uptake)

b NPV: negative predictive value.

c PPV: positive predictive value.

d AUC: area under the receiver operating characteristic curve.

The SMOTE results, including ROC curves and performance metrics measured at the Youden index are provided in Multimedia Appendix 1. The results did not show any improvement, and the overall performance significantly reduced. The best-performing model was the ensemble model with 73.63% AUC, which is lower than the AUC without SMOTE (ie, 79.61%). All other models demonstrated a notable decrease in AUC and other performance metrics, especially accuracy, specificity, and NPV.

**Figure 4.**
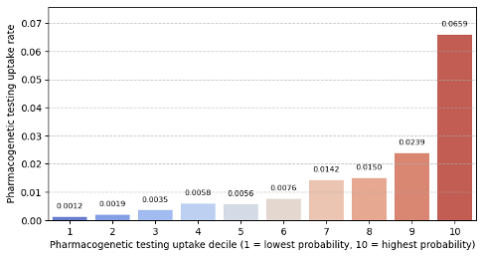
Stratification analysis for the probability of pharmacogenetic testing uptake.

Stratification analysis results are presented in Figure 4. The analysis was performed by dividing the predicted probabilities of pharmacogenetic testing uptake into deciles and calculating observed uptake rates within each to assess performance across the probability groups. The lowest decile was decile 1 with the lowest probability values, while decile 10 represented the highest probability values. Within each decile, observed uptake rates were calculated to plot against the probability deciles. The observed event rates demonstrated an increasing trend across the probability deciles (except for the 5th). The event rate in the highest probability decile was 6.59% (320/4853) while 0.12% (6/4853) in the lowest decile.

**Figure 5.**
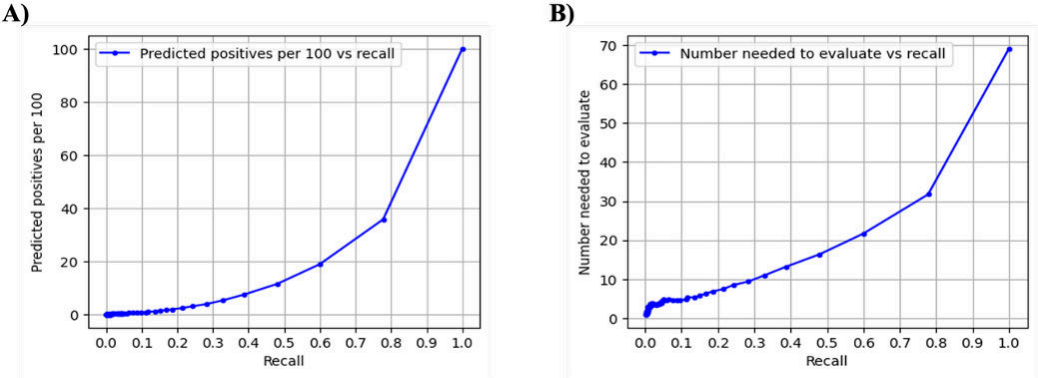
Recall versus (A) predicted positives per 100 and (B) number needed to evaluate.

Figure 5 demonstrates the plots of recall values against predictive positives per 100 and the number needed to evaluate. Predictive positives per 100 represent the number of individuals per 100 who are predicted to undergo pharmacogenetic testing across different recall values. The number needed to evaluate indicates the number of individuals that need to be assessed by the model to find 1 true positive (ie, recorded pharmacogenetic testing uptake).

**Figure 6.**
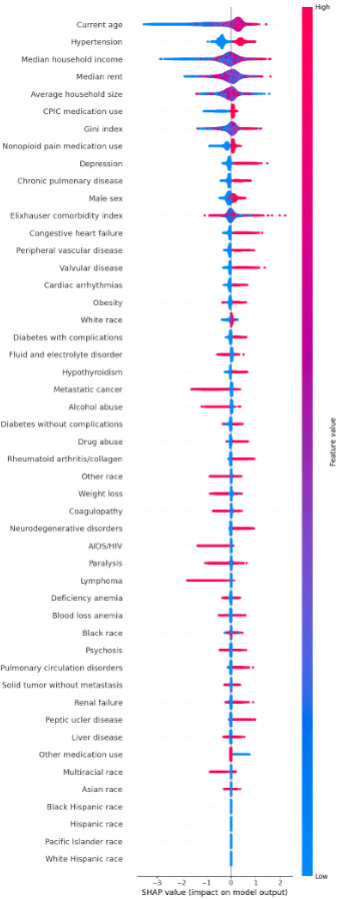
Shapley Additive Explanations (SHAP) analysis for feature importance (the x-axis shows the impact on prediction and the color represents the feature value, ie, red for high and blue for low). CPIC: Clinical Pharmacogenetics Implementation Consortium.

SHAP analysis for feature importance using the XGB model has been shown in Figure 6. Age had the highest impact on pharmacogenetic testing uptake, where older patients are more likely to undergo pharmacogenetic testing. Hypertension and median household income were the next most important features, both associated with a higher likelihood of pharmacogenetic testing uptake. Other SDoH factors were among the top 7 important factors for pharmacogenetic testing uptake. In contrast, racial group variables (ie, Hispanic, Pacific Islander, and White Hispanic) had the lowest influence on the outcome.

## Discussion

### Principal Findings

ML has widely been used in pharmacogenetics, where investigations focused on using genetic information to predict drug response [23,24]. However, this is the first study to develop ML models for predicting probability of the use of pharmacogenetic testing using clinical and nonclinical factors such as demographics, comorbidities, medication history, and SDoH. It highlighted the potential of using ML methods to improve the use of pharmacogenetic testing, especially in pain management with opioid medications. The results of this study demonstrated that the ensemble model achieved the highest AUC compared to other models for pharmacogenetic testing uptake prediction. Although the XGB model, when evaluated at its Youden index, performed better than other models in terms of accuracy, recall, specificity, and NPV, we selected the ensemble model as the final classifier. This was because the ensemble model harnesses the strengths of both high-performing XGB and SVM-RBF models for prediction, ultimately improving the performance for pharmacogenetic testing uptake prediction. Overall, the ensemble model proves to be a viable tool for accurately predicting pharmacogenetic testing uptake, supporting more informed, data-driven decision-making.

Several methodological considerations must be addressed when interpreting these promising results. The primary goal of our study was to predict pharmacogenetic testing uptake, which is a clinically favorable outcome. In this scenario, NPV represents the ability of the model to correctly predict no pharmacogenetic testing uptake out of all no-uptake predictions. While our study showed a very high NPV (32,162/32,327; 99.49%), a low NPV could lead to negative consequences, including resource misallocation, alert fatigue, and missed clinical opportunities for those who need pharmacogenetic testing uptake for more effective treatment. In contrast, PPV represents the accuracy of the model in correctly predicting pharmacogenetic testing uptake out of all positive predictions. Thus, a lower PPV leads to lower trustworthiness of positive predictions and inadequate resource allocation to patients who are wrongly predicted to undergo pharmacogenetic testing. Since our data was highly imbalanced with a low percentage of patients having a recorded pharmacogenetic testing uptake, we used a balanced class weighting approach for final model development, assigning proportionally higher weights to the minority class (ie, pharmacogenetic testing uptake). However, the model achieved a much lower PPV compared to NPV. To address this issue, threshold moving was used as a strategy to balance clinical context and consequences of false positives compared to false negatives. Since lowering and raising the threshold can decrease or increase PPV and NPV, the optimal threshold should be selected based on the trade-off between minimizing the consequences of misallocated interventions (ie, higher NPV) and maximizing clinical opportunities for patient care (ie, higher NPV). Overall, the high NPV in our study based on the Youden index could be advantageous in decision-making, as it allows for the allocation of more resources and encouragement plans for patients predicted not to undergo pharmacogenetic testing.

We conducted a comprehensive feature importance analysis to enhance model interpretability and clinical acceptance. The SHAP value analysis using the XGB model highlighted the contribution of each feature to individual predictions, enhancing the model’s clinical credibility and trustworthiness. The most influential features were age, hypertension, and household income, suggesting that these features require more attention for increasing pharmacogenetic testing uptake among patients. As mentioned, pharmacogenetic testing uptake is not necessarily due to opioid prescribing and could be due to prescribing of other medications and participation in a research project, potentially affecting the study results. Thus, although the results demonstrate the model’s transparency and clinical use, more research is needed before integrating the model into a real-world decision support system. While the SHAP diagram ranks features based on their overall importance, it is crucial to examine critical factors for each patient on a case-by-case basis when making decisions.

Beyond individual prediction, we evaluated the model’s ability to stratify patients into meaningful pharmacogenetic testing uptake categories. The uptake stratification analysis showed that the ensemble model effectively differentiated patients across pharmacogenetic testing uptake deciles, with higher uptake probabilities corresponding to higher observed uptake rates. Such an analysis can assist decision-makers in categorizing patients into different deciles based on their likelihood of pharmacogenetic testing uptake and prioritize those in lower deciles (less likely for the pharmacogenetic testing uptake) for optimal resource allocation and improved access to testing. It is important to recognize that stratification analysis often relies on predefined thresholds (ie, 10% in this study) to categorize probabilities into deciles, which may not be optimal for real-world decision-making.

The implementation of the pharmacogenetic testing uptake prediction model addresses the barriers to pharmacogenetic testing in opioid therapy. Our models accurately predict the likelihood of pharmacogenetic testing uptake but do not identify patients who clinically require or should receive testing (this was not the objective of this study). The models capture associations between demographic, clinical, and social determinants with testing behavior under current practice patterns, not clinical appropriateness. This distinction is important as the predictions reflect existing health care access and use patterns rather than evidence-based treatment recommendations. The predictive patterns we observed, favoring older patients with multiple comorbidities and greater health care access, largely reflect existing health care use behaviors and socioeconomic factors rather than clinical need or potential for improved outcomes. Consequently, this model should not be interpreted as a clinical decision support tool for determining which patients should receive pharmacogenetic testing. Rather, the primary applications of this model lie in health care operations and policy rather than direct clinical care. By providing the probability of pharmacogenetic testing uptake at the individual patient level, health systems can identify patients less likely to receive guideline-recommended testing and target barrier-reduction efforts. Insurers can use uptake predictions to forecast costs, allocate resources to underserved populations, and design coverage policies that promote equitable access to precision medicine. Such predictions could be particularly useful for patients predicted to benefit from testing (not the focus of this study), allowing care teams to prioritize resources to promote uptake in this population. By quantifying uptake likelihood, the model supports data-driven decisions about resource allocation and coverage policies to move beyond trial-and-error opioid prescribing toward more systematic, evidence-based pain management.

Our study has several limitations. First, it is notable that although the study used PPV as a performance metric, it is highly dependent on outcome prevalence and may not generalize across different patient populations or clinical settings, reducing its applicability in decision-making. Second, this study used various clinical and demographic variables to develop more-informed ML models. However, the underrepresentation of certain racial groups, such as Hispanic and Pacific Islander, could potentially affect models’ outcomes. This raises concerns about the bias and fairness issues, as ML models may exhibit lower performance for these groups compared to others. Third, while the study incorporated SDoH information as potential predictors of pharmacogenetic testing uptake, this information was based on ZIP code-level data, which may lack the necessary granularity required for precise prediction. Detailed individual-level data on variables such as income, education, and household size could enhance the model’s reliability and improve the accurate identification of each feature’s contribution to the outcome. Fourth, the model’s performance may not directly translate to external populations with varying demographic and clinical backgrounds. Specifically, the model was trained on data from a single EHR system, including minority racial groups, and the results may not be applicable to other racial groups. Therefore, caution is warranted when interpreting the model’s outcomes for these populations. Finally, some of the patients in the data who had a recorded pharmacogenetic testing uptake were part of a clinical trial where the cost of testing was covered. This would potentially influence the ability of the model to identify the true impact of patients’ economic and income status on undergoing pharmacogenetic testing. To demonstrate the model’s broader applicability, future work should focus on external validation using independent datasets from different health systems and identification of patients who are more likely to benefit from testing. This would help evaluate the model’s robustness and clinical use for real-world decision-making.

### Conclusions

Pharmacogenetic testing is a viable tool for matching patients’ genetic profiles to suitable opioids for pain treatment. This study proposed ML models for pharmacogenetic testing uptake prediction using data from an EHR system. Results demonstrated that the ensemble ML model combining XGB and SVM-RBF classifiers achieved the highest AUC at 79.61%, making it a reliable prediction model for pharmacogenetic testing uptake prediction. Additionally, the uptake stratification and feature importance analysis using SHAP values further indicated the model’s use for real-world applications. Following further validation using an external dataset, this model can be integrated into a data-driven decision support system, enabling health systems and insurers in resource planning and health equity assessment to optimize pain management and improve patient outcomes.

## Supplementary material

10.2196/81048 Multimedia Appendix 1 Synthetic minority over-sampling technique results.

## Abbreviations

AUC: area under the receiver operating characteristic curve
CPIC: Clinical Pharmacogenetics Implementation Consortium
CYP2D6: cytochrome P450 family 2 subfamily D member 6
EHR: electronic health record
*ICD-10*: *International Classification of Diseases, Tenth Revision*
*ICD-9*: *International Classification of Disease, Ninth Revision*
IDR: Integrated Data Repository
LR: logistic regression
LSVM: linear support vector machine
ML: machine learning
NB: naïve Bayes
NN: neural network
NPV: negative predictive value
PPV: positive predictive value
RF: random forest
SDoH: social determinants of health
SHAP: Shapley Additive Explanations
SMOTE: synthetic minority over-sampling technique
SVM: support vector machine
SVM-RBF: support vector machine–radial basis function
TRIPOD+AI: Transparent Reporting of a Multivariable Prediction Model for Individual Prognosis or Diagnosis + Artificial Intelligence
XGB: extreme gradient boosting
