# Supplementary material for: Machine Learning Prediction of Pharmacogenetic Testing Uptake Among Opioid-Prescribed Patients Using Electronic Health Records: Retrospective Cohort Study
Source: JMIR Med Inform. 2026 Jan 21;14:e81048. doi: 10.2196/81048 (PMC12822862; doi:10.2196/81048)
Supplement: Multimedia Appendix 1 [file medinform-v14-e81048-s001.docx]

**Appendix 1.** SMOTE results.

***
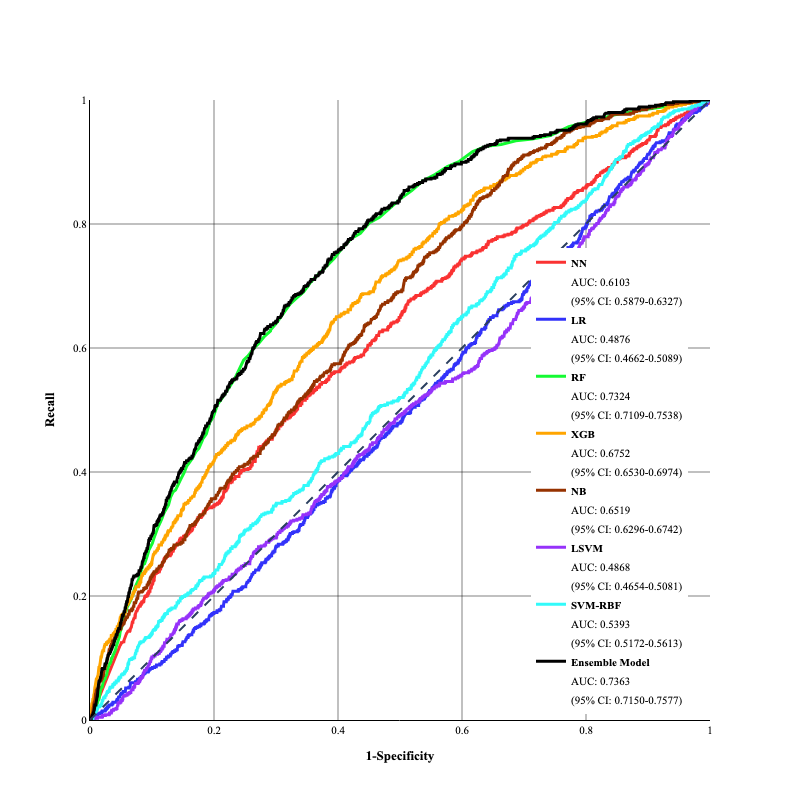
***

**Fig. 1.** ROC curves of developed models after SMOTE.

**Table 1.** Pefromance metrics of developed models after SMOTE based on Youden index.

| **Model** | **Accuracy** | **Recall** | **Specificity** | **NPV** | **PPV** | **AUC** |
| --- | --- | --- | --- | --- | --- | --- |
| NN | 67.76 | 48.71 | **68.04** | 98.91 | 2.18 | 61.03 |
| LR | 9.93 | 92.98 | 8.72 | 98.84 | 1.46 | 48.76 |
| RF | 58.14 | 77.65 | 57.85 | 99.44 | 2.62 | 73.24 |
| XGB | 59.68 | 65.19 | 59.60 | 99.15 | 2.30 | 67.52 |
| NB | 32.47 | 89.83 | 31.64 | **99.53** | 1.88 | 65.19 |
| LSVM | 5.06 | **97.42** | 3.71 | 99.00 | 1.46 | 48.68 |
| SVM-RBF | 32.24 | 74.36 | 31.63 | 98.83 | 1.56 | 53.93 |
| Ensemble | **60.65** | 75.36 | 60.44 | 99.41 | **2.70** | **73.63** |
